# Repair and DNA Polymerase Bypass of Clickable Pyrimidine Nucleotides

**DOI:** 10.3390/biom14060681

**Published:** 2024-06-12

**Authors:** Anton V. Endutkin, Anna V. Yudkina, Timofey D. Zharkov, Alexander E. Barmatov, Daria V. Petrova, Daria V. Kim, Dmitry O. Zharkov

**Affiliations:** 1 Siberian Branch of the Russian Academy of Sciences Institute of Chemical Biology and Fundamental Medicine, 8 Lavrentieva Ave., 630090 Novosibirsk, Russia; ayudkina@niboch.nsc.ru (A.V.Y.); timazharkov74@gmail.com (T.D.Z.); poslanie01@gmail.com (A.E.B.); dpetrova@niboch.nsc.ru (D.V.P.); dkim@niboch.nsc.ru (D.V.K.); 2 Department of Natural Sciences, Novosibirsk State University, 2 Pirogova St., 630090 Novosibirsk, Russia

**Keywords:** click chemistry, metabolic labeling, DNA repair, replication, DNA glycosylases, DNA polymerases, 5-ethynyl-2′-deoxyuridine

## Abstract

Clickable nucleosides, most often 5-ethynyl-2′-deoxyuridine (EtU), are widely used in studies of DNA replication in living cells and in DNA functionalization for bionanotechology applications. Although clickable dNTPs are easily incorporated by DNA polymerases into the growing chain, afterwards they might become targets for DNA repair systems or interfere with faithful nucleotide insertion. Little is known about the possibility and mechanisms of these post-synthetic events. Here, we investigated the repair and (mis)coding properties of EtU and two bulkier clickable pyrimidine nucleosides, 5-(octa-1,7-diyn-1-yl)-U (C8-AlkU) and 5-(octa-1,7-diyn-1-yl)-C (C8-AlkC). In vitro, EtU and C8-AlkU, but not C8-AlkC, were excised by SMUG1 and MBD4, two DNA glycosylases from the base excision repair pathway. However, when placed into a plasmid encoding a fluorescent reporter inactivated by repair in human cells, EtU and C8-AlkU persisted for much longer than uracil or its poorly repairable phosphorothioate-flanked derivative. DNA polymerases from four different structural families preferentially bypassed EtU, C8-AlkU and C8-AlkC in an error-free manner, but a certain degree of misincorporation was also observed, especially evident for DNA polymerase β. Overall, clickable pyrimidine nucleotides could undergo repair and be a source of mutations, but the frequency of such events in the cell is unlikely to be considerable.

## 1. Introduction

Accelerated by the discovery of Cu(I)-catalyzed azide–alkyne cycloaddition in the early 2000s, click chemistry reactions quickly matured into a valuable tool for molecular and cell biology research and drug discovery [1,2]. Among a slew of other applications, click chemistry found a special niche in studies of DNA metabolism in living cells due to the excellent substrate properties of some clickable dNTPs for DNA polymerases [3,4,5]. Cell treatment with alkyne-carrying clickable nucleosides, most often 5-ethynyl-2′-deoxyuridine (EtU), allows for the vital labeling of newly replicated DNA, which can be later visualized after conjugation with a fluorescent dye equipped with an azide moiety. In addition to metabolic labeling, clickable nucleosides are finding use in DNA functionalization for making conjugates with other nucleic acids, proteins and other molecules both in vitro and in vivo with a wide array of linkers connecting the alkyne group and the nucleobase [4,6]. Commonly, functional groups in such modifications are introduced at the C5 position of the uracil ring due to the easy synthetic availability of 5-iodo-dU, a common precursor of 5-substituted derivatives, and 5-azidomethyl-dU, 5-vinyl-dU and 2′-fluoro-5-ethynyl-dU were also reported as cell labeling reagents [4,5]. However, clickable variants of other deoxynucleosides, both natural such as dC and unnatural such as 7-deazapurine-2′-deoxynucleosides, have been synthesized and used.

Once in the intracellular environment, either metabolically incorporated or delivered as part of synthetic constructs, clickable nucleobases in DNA should come under the surveillance of DNA repair and DNA damage response systems, which recognize any modification, except for natural epigenetic ones like 5-methylcytosine or 5-hydroxymethylcytosine, as a potential lesion [7,8]. Several partially overlapping DNA repair mechanisms have been characterized in human cells, of which damaged bases are dealt with by base excision repair (BER), nucleotide excision repair (NER), mismatch repair and direct reversal pathways [7,9,10]. Many C5-modified pyrimidines, such as 5-halogenated ones also widely used in vital DNA labeling, as well as 5-hydroxymethyluracil, 5-formyluracil, 5-formylcytosine, 5-carboxyluracil and 5-carboxylcytosine, are quite efficiently removed via the BER pathway [11,12,13,14,15,16,17,18]. If not repaired, modified nucleosides in DNA may be encountered by DNA polymerases in the next round of replication or during the repair of the opposite strand, which may result in cytotoxic polymerase stalling or mutagenic bypass. The latter is facilitated by specialized error-prone translesion DNA polymerases that protect cells from replication fork collapse-induced death at the expense of generating mutations in a process called DNA damage tolerance [19,20].

**Figure 1:**
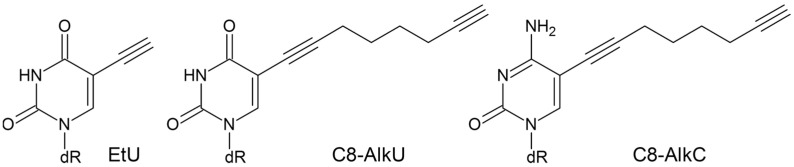
Structures of clickable U and C derivatives investigated in this work.

Little is known about the repairability of clickable nucleotides or their interactions with DNA polymerases. This problem is admitted in the field, as removal by repair is one recognized source of label disappearance in long-term experiments that cannot be reliably controlled for at present [5]. In a recent report, EtU was shown to be removed from genomic DNA by a transcription-coupled branch of NER in human fibroblasts [21]; however, other repair options remain unexplored. Given the ability of some DNA glycosylases to excise C5-modified pyrimidines, BER might also play a role in the processing of clickable nucleotides. Here, we addressed the possibility of glycosylase-initiated repair, polymerase stalling and (mis)coding for EtU, the most popular clickable nucleoside, and two pyrimidine nucleosides with an alkyne group connected through a longer linker, 5-(octa-1,7-diyn-1-yl)-U (C8-AlkU) and 5-(octa-1,7-diyn-1-yl)-C (C8-AlkC), which are used for the functionalization of synthetic DNA [22] (Figure 1).

## 2. Materials and Methods

### 2.1. Enzymes and Oligonucleotides

Human UNG (catalytic domain, residues 85–304 of the UNG1 isoform) [23], SMUG1 [24], MBD4 (catalytic domain, residues 426–580) [18], NEIL1 [25], NEIL2 [26], OGG1 [27], DNA polymerases β (POLβ) [28], κ (POLκ; catalytic domain, residues 1–560) [29] and λ (POLλ; catalytic domain, residues 242–575) [30], Klenow fragment of *Escherichia coli* DNA polymerase I (KF, exonuclease-deficient D355A/E357A mutant) [31] and phage RB69 DNA polymerase (RBpol, exonuclease-deficient D222A/D327A mutant) [32] were purified using the protocols described in the papers referenced above. The data on the purity and activity of the proteins are available in supplementary information to Ref. [28]. All oligonucleotides (Supplementary Table S1) were synthesized in-house from commercially available phosphoramidites (Glen Research, Sterling, VA, USA). If necessary, the oligonucleotides were 5′-labeled using phage T4 polynucleotide kinase (SibEnzyme, Novosibirsk, Russia) and γ[^32^P]ATP (ICBFM Laboratory of Biotechnology, Novosibirsk, Russia). Primers for DNA polymerase reactions carried a synthetically introduced 5(6)-carboxyfluorescein label at the 5′-end.

### 2.2. DNA Glycosylase Assays

The reaction mixture (10 μL) included 50 mM Tris–HCl (pH 7.5), 100 mM NaCl, 1 mM Na ethylenediaminetetraacetate (EDTA), 1 mM dithiothreitol (DTT), 50 nM double-stranded DNA substrate and 1 µM (total protein) enzyme. The mixtures were incubated for 1 h at 37 °C and quenched by adding NaOH to 0.1 M and heating for 3 min at 95 °C. The samples were neutralized with an equimolar amount of HCl and mixed with 0.5× volume of gel-loading solution (20 mM EDTA, 0.1% xylene cyanol, 0.1% bromophenol blue in 80% formamide). The reaction products were resolved by denaturing 20% polyacrylamide gel electrophoresis and quantified by phosphorimaging (Typhoon FLA 9500, GE Healthcare, Chicago, IL, USA).

### 2.3. Singe-Turnover Kinetics of MBD4 or SMUG1

The reaction mixture (50 μL) included the reaction buffer as described above, 50 nM DNA duplex containing etU, C8AlkU, U or T (with G opposite to the lesion) and 1 µM MBD4 or SMUG1 (total protein). The reactions were allowed to proceed for 0–60 min at 37 °C; within this time period, 5 µL aliquots were withdrawn and treated as described above. The apparent rate constant of base excision, *k*_2 app_, was determined from three independent experiments by fitting the data to the equation $\left[P\right]=A\left(1-e^{-k_{2app}t}\right)$, where [P] is product concentration, *A* is the maximal cleavage and *t* is time, using SigmaPlot v11.0 (Grafiti, Palo Alto, CA, USA).

### 2.4. DNA Polymerase Assays

To prepare DNA substrates, a fluorescently labeled primer (top12, Supplementary Table S1), a template strand (23EtU, 23C8-AlkU, or 23C8-AlkC) and, if necessary, a 5′-phosphorylated downstream strand (top10) were annealed in a 1:2:2 molar ratio. The reaction mixture (10 µL) contained 100 nM DNA substrate, 500 µM dNTP (either dATP, dTTP, dGTP or dCTP individually or their equimolar mixture amounting to 500 µM in sum) and one of the following polymerases: KF (2 nM), RBpol (2 nM), POLβ (5 nM), POLλ (10 nM) or POLκ (5 nM) in the buffer consisting of 50 mM Tris–HCl (pH 7.5, or pH 8.5 for POLλ), 1 mM DTT and 5 mM MgCl_2_ (or 10 mM for POLβ or 2.5 mM for POLλ). The reaction was allowed to proceed for 10 min at 25 °C and quenched with an equal volume of 10 mM EDTA in formamide and heating at 95 °C for 2 min. The products were separated and visualized as described above.

### 2.5. Steady-State DNA Polymerase Kinetics

The reaction conditions and treatment were identical to those described above, except the concentrations of dNTP were varied within the 2 µM–1000 µM range. The concentration of each DNA polymerase was adjusted for each substrate to give less than a 20% incorporation of the first dNMP (Supplementary Table S2). The observed reaction velocities as a function of dNTP concentration were fitted by nonlinear regression to the Michaelis–Menten equation using SigmaPlot v11.0. All reported constants are derived from three to four independent experiments.

### 2.6. Reporter Plasmid Experiments

Plasmids pZAJ_Q205* carrying modified or unmodified nucleotides in the *eGFP* gene were generated as previously described [33,34]. HeLa cells were seeded in 12-well plates at 10^5^/well density, grown for 24 h and transfected with 100 ng of the construct and 300 ng of pDsRed-Monomer-N1 (Takara Bio, Kusatsu, Japan) using the Effectene reagent (Qiagen, Venlo, The Netherlands). The cells were collected 24 h post-transfection and analyzed on a Novocyte 3000 flow cytometer (Agilent Technologies, Santa Clara, CA, USA). For data analysis, the median green fluorescence of EGFP^+^ DsRed^+^ cells transfected with pZAJ_Q205* was normalized for the median green fluorescence of EGFP^+^ DsRed^+^ cells transfected with the pZAJ_5c plasmid, which carries wild-type *eGFP*; the normalization and gating strategies are described in [35,36,37]. Two-tailed Student’s *t*-test with Bonferroni correction was used to compare the groups.

## 3. Results

### 3.1. SMUG1 and MBD4 DNA Glycosylases Can Excise Clickable Pyrimidine Bases

We reasoned that if clickable pyrimidine derivatives can be removed by DNA repair, this might likely involve the base excision repair (BER) pathway [38,39]. In its most common form, BER is initiated by one of several DNA glycosylases, which recognize a specific subtype of damaged bases and hydrolyze their *N*-glycosidic bond. Then, the resulting abasic (AP, for apurinic/apyrimidinic) site is cleaved by an AP endonuclease to produce a 3′-OH terminus amenable to extension by DNA polymerases and a 5′-terminal 2′-deoxyribose-5′-phosphate. Alternatively, some DNA glycosylases catalyze the elimination of the 3′-phosphate after base excision (AP lyase reaction), and the residual α,β-unsaturated 3′-terminal deoxyribose remnant is removed by the phosphodiesterase activity of AP endonucleases. The repair is followed by the incorporation of one or several dNMPs, the processing of the downstream strand and ligation. In human cells, the BER pathway repairs mostly small non-distorting lesions, such as those caused by nucleobase oxidation, deamination, alkylation or spontaneous base loss but also can deal with more complex damage including intrastrand cross-links and some types of bulky adducts [38,39,40].

To address the possibility of the recognition of clickable pyrimidine bases by BER, we screened a panel of human DNA glycosylases for their ability to excise EtU, C8-AlkU and C8-AlkC bases. We assayed UNG, SMUG1, MBD4 (also known as MED1), NEIL1, NEIL2 and OGG1 proteins. UNG, SMUG1 and MBD4 are glycosylases specific for uracil and can process some of its C5 derivatives, especially MBD4, which efficiently removes T from T:G mispairs [41,42]. It should also be noted that MBD4 predominantly recognizes lesions in the CpG context (e.g., if C or 5-methylcytosine are deaminated to U or T, respectively) [41,42]. NEIL1 and NEIL2 can release various kinds of modified pyrimidines and fragmented purines, mostly oxidatively damaged ones [43]. The major substrate of OGG1 is 8-oxoguanine and formamidopyrimidines [44]. Considering that normally C5-modified pyrimidines would be incorporated opposite A (uracil derivatives) or G (cytosine derivatives), we tested the removal of clickable pyrimidine bases from oligonucleotide duplexes paired with A or G and also from single-stranded (ss) DNA.

**Figure 2:**
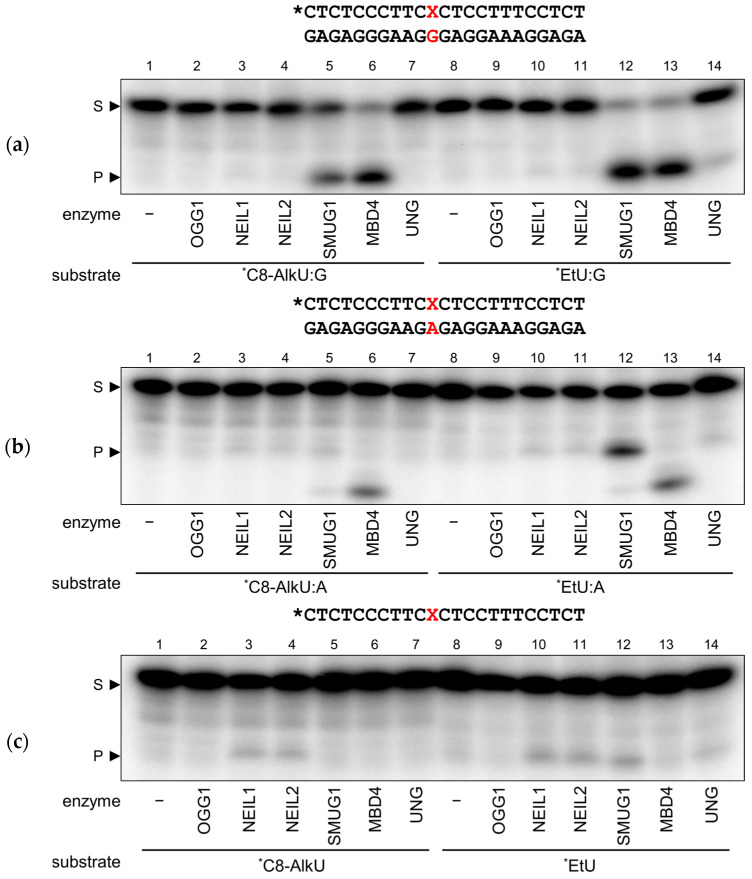
The cleavage of DNA substrates containing C8-AlkU or EtU paired with G (**a**) or A (**b**) or in single-stranded DNA (**c**) by DNA glycosylases. S, substrate; P, cleavage product. The asterisk indicates the ^32^P-labeled strand. The structures of the substrates are shown above the gel images; X, modified base. Original images of (**a**–**c**) can be found in Supplementary Materials.

Of the studied enzymes, only SMUG1 and MBD4 were able to process both EtU:G- and C8-AlkU:G-containing substrates (Figure 2a). UNG gave only trace amounts of the product at best, while NEIL1, NEIL2 and OGG1 yielded no cleavage despite the long reaction time and a 20-fold enzyme excess. EtU:A, but not C8-AlkU:A, was also processed by SMUG1 albeit with much lower efficiency (Figure 2b). None of the tested glycosylases significantly excised EtU or C8-AlkU from single-stranded substrates (Figure 2c). C8-AlkC was refractory to cleavage in all cases (Supplementary Figure S1). MBD4 also yielded some slow non-specific cleavage of EtU:A, C8-AlkU:A, C8-AlkC:A and ssC8-AlkC substrates (high-mobility band in Figure 2b); at the moment, we did not pursue the nature of the product.

**Figure 3:**
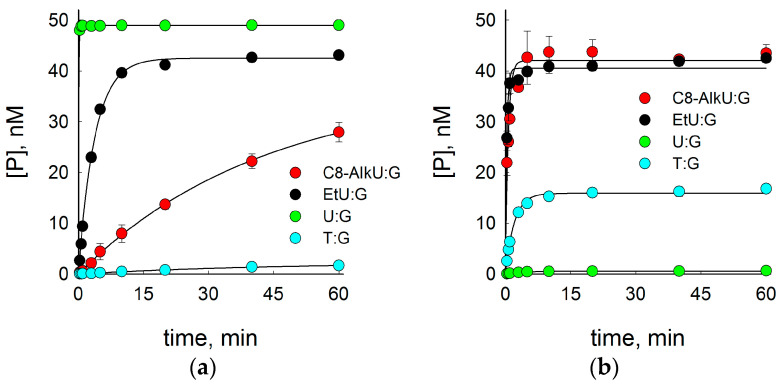
Time course of product accumulation under single-turnover conditions during substrate cleavage by SMUG1 (**a**) and MBD4 (**b**). Circles and error bars indicate mean ± s.d. of three independent experiments; lines are fits to equation $\left[P\right]=A\left(1-e^{-k_{2app}t}\right)$ (see Section 2.3). Black symbols, EtU:G substrate; red, C8-AlkU:G; green, U:G; cyan, T:G.

For a more quantitative characterization of the SMUG1 and MBD4 activity on EtU and C8-AlkU substrates, we compared it with the activity on U:G and T:G in single-turnover kinetic experiments (Figure 3, Table 1). Both SMUG1 and MBD4 are slow-turnover enzymes [45,46], so their regular steady-state kinetics are limited by the product release step and provides little information on their catalytic efficiency. Taking the enzyme in a large excess allows one to compare their apparent catalytic constants, *k*_2 app_, for different substrates. SMUG1, consistent with the literature reports [15], very efficiently removed U from U:G pairs and also processed T:G pairs but conducted it very slowly (Figure 3a, Table 1). In fact, at the first analyzed moment of the time course (20 s), the cleavage of the U:G substrate was almost complete, so the *k*_2 app_ value of 12 min^−1^ reported in Table 1 is a lower estimate. In comparison, SMUG1 processed EtU:G ~2 orders of magnitude slower than U:G but approximately 11-fold more efficiently than C8-AlkU:G (Figure 3, Table 1).

The situation with MBD4 was drastically different: both U:G and T:G were cleaved much less efficiently than EtU:G and C8-AlkU:G (Figure 3b, Table 1). While the low activity on U:G and T:G might be due to the placement of the lesion or the mismatch in a non-CpG sequence, the robust excision of both clickable nucleobases indicates that the CpG context is not strictly necessary for the activity of MBD4 (or at least of its catalytic domain) on some substrate lesions.

### 3.2. Normal Coding and Miscoding by Clickable Pyrimidine Bases

To analyze the behavior of a representative selection of DNA polymerases, we chose the Klenow fragment of *E. coli* DNA polymerase I (KF), phage RB69 DNA polymerase (RBpol) and human DNA polymerases β, λ and κ. KF is probably the best-studied DNA polymerase in terms of its biochemistry; it belongs to structural Family A along with human DNA polymerases γ, θ and ν [47]. RBpol represents structural Family B, which also includes the main human replicative polymerases α, δ and ε, and translesion DNA polymerase ζ [47,48]. KF and RBpol are often used as convenient substitutes to study the enzyme mechanisms presumably conserved in their less accessible human counterparts. POLβ and POLλ come from Family X, the members of which are mostly involved in DNA repair, others being DNA polymerase μ and terminal deoxynucleotidyl transferase [47,49,50,51]. Finally, POLκ is a translesion DNA polymerase from Family Y, participating in the DNA damage tolerance pathway together with DNA polymerases η, ι and REV1 [47,51,52]. Together, the five polymerases studied here represent all DNA-dependent DNA polymerase families encoded in the human genome with the exception of primase–polymerase.

**Figure 4:**
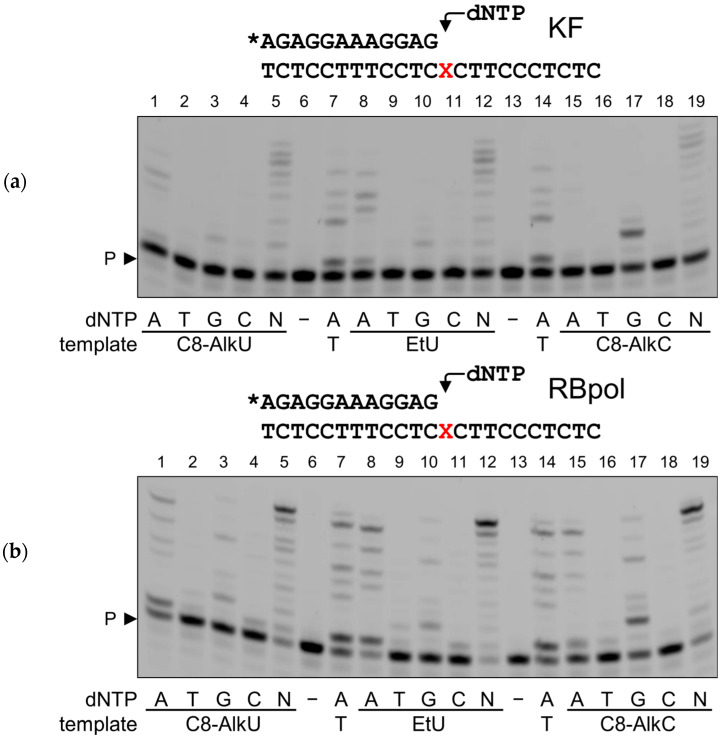
Primer extension by KF (**a**) and RBpol (**b**) across EtU, C8-AlkU and C8-AlkC. The template modification and dNTPs are indicated under the gel images. N, an equimolar mixture of all four dNTPs. P, primer. The structures of the substrates and the nature of the polymerase are shown above the gel images; X, modified base; the asterisk indicates the fluorescein-labeled primer. Original images of (**a**,**b**) can be found in Supplementary Materials.

Having encountered EtU in the template, KF mostly behaved identically to the regular dAMP opposite to T incorporation (Figure 4a, lanes 8–12). The same was true for the C8-AlkU template, but the reaction was somewhat less efficient (Figure 4a, lanes 1–5). A minor incorporation of dGMP was also observed (Figure 4a, lanes 3, 10). Only dGMP was incorporated opposite to C8-AlkC. Highly processive polymerases such as KF and RBpol are noted for their ability to induce dNTP-stabilized primer–template misalignment [53,54], which can be seen as the appearance of multiple extension products with dATP both with the EtU and C8-AlkU templates and in the control reaction.

RBpol demonstrated a similar but more promiscuous behavior. While dAMP was preferably incorporated opposite to EtU and C8-AlkU, and dGMP, opposite to C8-AlkC (Figure 4b), the non-specific incorporation of dGMP opposite to EtU and C8-AlkU and of dAMP opposite to C8-AlkC was much more pronounced. Moreover, a trace to minor incorporation of dCMP and dTMP was also observed. Considering dGMP incorporation by both RBpol and KF, the mobility of the product clearly corresponds to the extension of the primer by two nucleotides, meaning that the first one was misincorporated opposite to EtU or C8-AlkU, and then the second one was directed by C in the template (see Supplementary Table S1 for the template sequences). Presumably, the misinsertions are facilitated by the strong affinity of highly processive polymerases for the primer end and the lack of the proofreading activity in the exonuclease-deficient enzyme versions used here. Thus, both KF and RBpol bypass the clickable pyrimidine nucleotides rather efficiently but in a somewhat error-prone manner, at least under the experimental conditions used.

**Figure 5:**
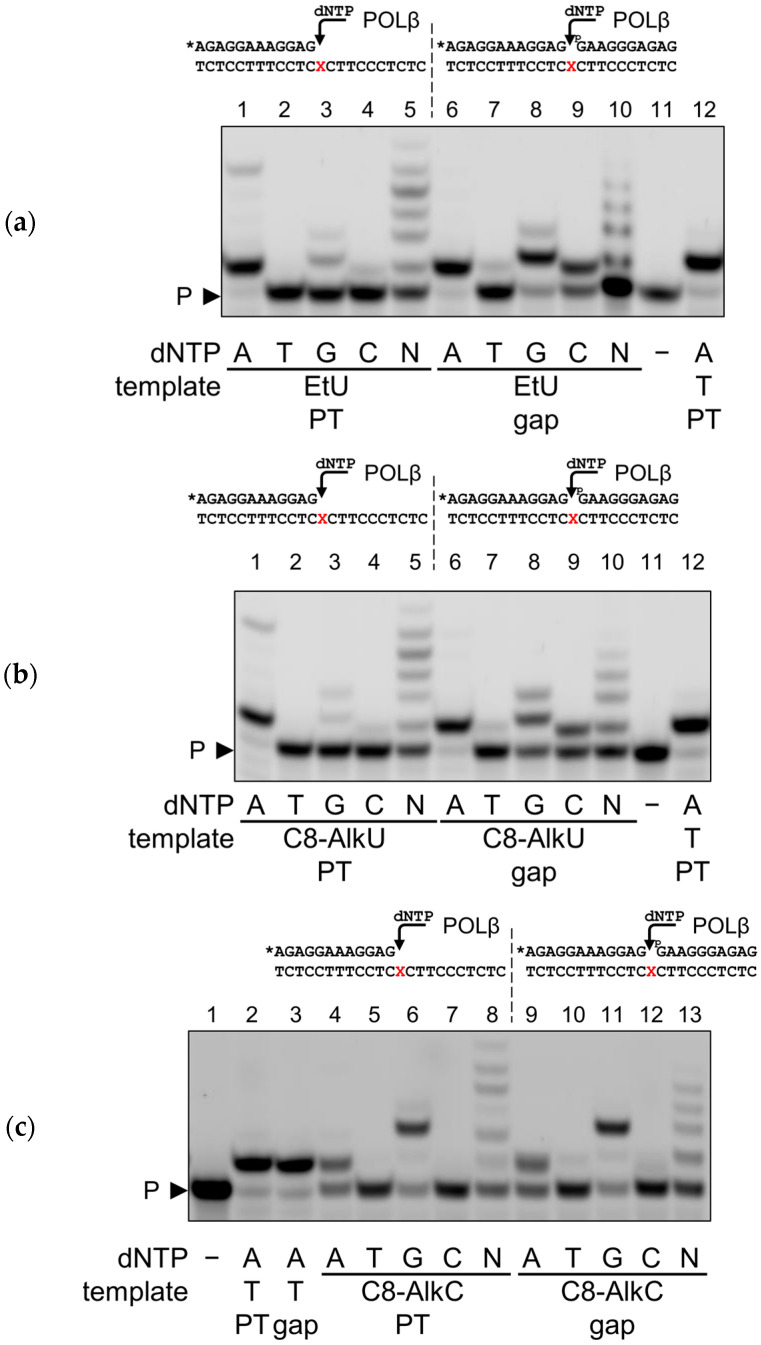
Primer extension by POLβ on the primer–template and gapped substrates across EtU (**a**), C8-AlkU (**b**) and C8-AlkC (**c**). The template modification and dNTPs are indicated under the gel images. N, an equimolar mixture of all four dNTPs. P, primer. The structures of the substrates and the nature of the polymerase are shown above the gel images; X, modified base; the asterisk indicates the fluorescein-labeled primer. PT, primer–template substrate; gap, 1 nt gap substrate. Original images of (**a**–**c**) can be found in Supplementary Materials.

Among other DNA polymerases, POLβ is known for its preference for substrates containing a single-nucleotide (1-nt) gap with a phosphorylated 5′-end of the downstream strand, which mimics the BER intermediate before the correct dNMP insertion [50]. Thus, for POLβ, we compared side-by-side the coding properties of EtU, C8-AlkU and C8-AlkC in the primer–template and 1 nt gap context (Figure 5). To our surprise, the gap clearly reduced the fidelity of POLβ, greatly increasing the incorporation of dGMP and dCMP opposite to EtU and C8-AlkU (Figure 5a,b). In contrast, dAMP was incorporated opposite to C8-AlkC with a similar efficiency both in the primer–template and the 1 nt gap substrate (Figure 5c).

**Figure 6:**
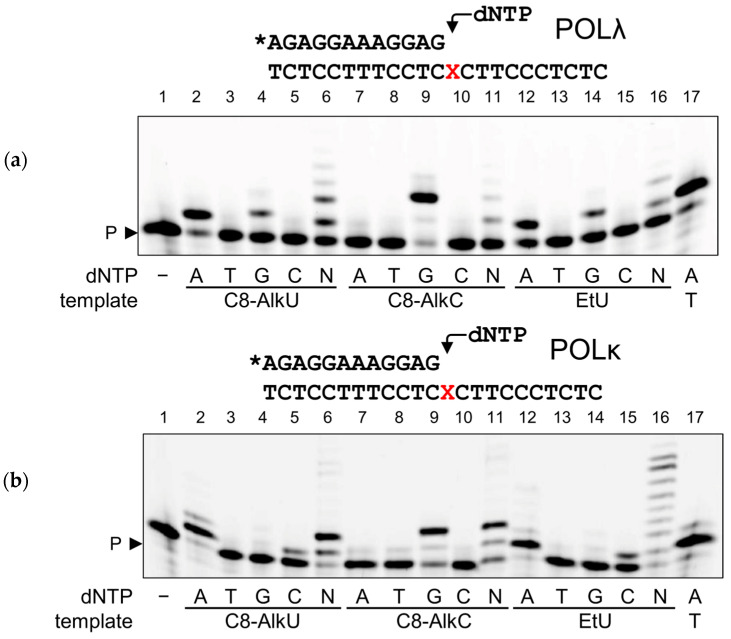
Primer extension by POLλ (**a**) and POLκ (**b**) across EtU, C8-AlkU and C8-AlkC. The template modification and dNTPs are indicated under the gel images. N, an equimolar mixture of all four dNTPs. P, primer. The structures of the substrates and the nature of the polymerase are shown above the gel images; X, modified base; the asterisk indicates the fluorescein-labeled primer. Original images of (**a**,**b**) can be found in Supplementary Materials.

POLλ, a paralog of POLβ, is mainly involved in non-homologous end-joining where it fills in short resected ends of double-strand breaks [55,56]. POLλ also preferentially incorporated a complementarity-directed nucleotide, i.e., dAMP for EtU and C8-AlkU and dGMP for C8-AlkC in the template, together with a minor incorporation of dGMP opposite to EtU and C8-AlkU (Figure 6a). No misincorporation opposite to C8-AlkC was observed. POLκ, on the other hand, displayed a peculiar error pattern: while it also preferred complementary dNMP, POLκ was the only one of the studied polymerases to misincorporate dCMP much better than dGMP opposite to EtU and C8-AlkU (Figure 6b). Also, the extension by POLκ beyond 2 nt was very strongly suppressed by long linkers in C8-AlkU and C8-AlkC (compare lanes 6, 11 and 16 in Figure 6b), consistent with the general tendency of major groove adducts to inhibit this polymerase [52].

We then carried out a limited steady-state kinetic characterization of RBpol, as a homolog of human replicative DNA polymerases and POLβ as the major human DNA repair polymerase (Table 2). Based on the specificity constant, *k*_cat_/*K*_M_, RBpol incorporated the complementary nucleotide opposite to C8-AlkC more efficiently than opposite to EtU, while C8-AlkU was apparently the worst substrate for which enzyme saturation by dNTP could not be achieved. Compared with the literature data for normal dNTPs and unmodified templates (*k*_pol_/*K*_d_ = 2.4–6.4 µM^−1^s^−1^) [48], the catalytic proficiency of RBpol on the clickable templates was reduced by at least two orders of magnitude, although part of this decrease might be due to a different substrate, reaction conditions and another type of kinetic assay (steady-state vs. single-turnover). POLβ used templates with all kinds of clickable nucleotides with a similar efficiency within the same type of substrate, and gapped substrates were clearly preferred, with *k*_cat_/*K*_M_ increasing ~5–8-fold for complementary dNTPs. Again, the efficiencies were greatly reduced compared with the reported values for primer extension on unmodified templates (0.3 µM^−1^s^−1^ and 4.4 µM^−1^s^−1^ for primer–template and gapped substrates, respectively [57]). The error frequency associated with dGMP and dCMP misincorporation opposite to EtU and C8-AlkU in the gapped substrates was within 1.4–5.6%.

### 3.3. Impaired Repair of Clickable Pyrimidine Bases in Human Cells

Finally, to evaluate the efficiency of clickable nucleotides’ removal from DNA in living cells, we used the reporter plasmid encoding a non-fluorescent variant of enhanced green fluorescent protein (EGFP) [58,59]. In this system, the reporter gene is disrupted by a TAG stop codon due to a c.613C>T mutation. The stop codon is flanked by two Nb.Bpu10I nickase recognition sites so that an 18 nt fragment can be excised from the non-coding strand and replaced with a synthetic oligonucleotide carrying a modification. During the readthrough by RNA polymerase II, the incorporation of any rNMP other than UMP produces RNA without the stop codon, which is then translated into a fluorescent EGFP variant (wild-type, Q205E or Q205K). However, the removal of the modified nucleotide and repair that uses the coding strand as a template restores the truncated EGFP [58,59].

**Figure 7:**
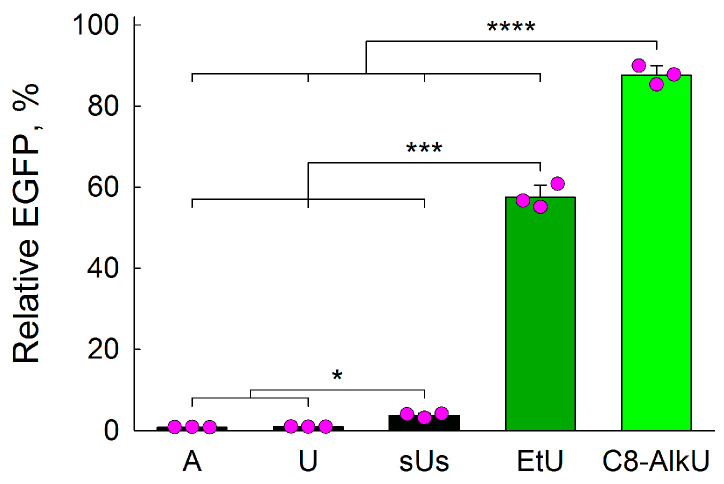
Transcriptional mutagenesis and repair of EtU and C8-AlkU in HeLa cells. Relative EGFP expression normalized for the fluorescence of the control G-construct is presented (*n* = 3, mean ± SD shown). *p* < 0.05 (*); *p* < 0.005 (***); *p* < 0.001 (****), two-tailed Student’s *t*-test with Bonferroni correction.

Since C8-AlkC was resistant to BER (Section 3.1), we limited the cell study to EtU and C8-AlkU. EtU seems to be only moderately blocking for human and *E. coli* RNA polymerases [21,60,61], so sufficient bypass to detect the fluorescent protein-encoding transcript was expected. As a positive control for repair, we used U, a lesion that is efficiently removed from DNA by UNG, SMUG1 and MBD4 [39]. Indeed, when U-carrying plasmids were transfected into HeLa cells, the fluorescence was not significantly different from that imparted by the parent construct with A opposite to T of the stop codon (Figure 7). When both phosphodiester bonds around dU were replaced with phosphorothioate linkages (sUs modification), which inhibit strand cleavage by AP endonucleases and AP lyases [62,63,64], the fluorescence signal was elevated significantly, although the magnitude of this increase was only about 4.2-fold. These results are consistent with our earlier observations in HEK293 cells [34] and suggest the existence of a still-to-be-identified backup repair pathway for genomic uracil. However, the outcome for EtU and C8-AlkU was strikingly different. The population of fluorescent cells increased, relative to sUs, 15-fold for EtU and 24-fold for C8-AlkU, and compared with easily repaired U, the rise was 62- and 94-fold, respectively (Figure 7). Since the plasmid we used does not replicate in HeLa cells [35], these findings suggest that EtU and C8-AlkU are quite resistant to removal in the absence of replication.

## 4. Discussion

While clickable nucleosides, most prominently EtU, are now widely used to follow nucleic acids’ metabolism in living cells, and a variety of those are available for making various kinds of DNA nanostructures and conjugates [3,4,5,6,22], surprisingly little is known about the interaction of these modifications, once incorporated, with enzymes involved in DNA repair and DNA synthesis. EtU was reported to be mutagenic and moderately genotoxic when administered as a deoxynucleoside to Chinese hamster ovary cells, with the toxicity more pronounced in the cells deficient in homologous recombination [65], suggesting at least some impairment of DNA polymerase action and the concomitant repair. Sancar and colleagues have recently shown that transcription-coupled NER excises EtU-containing fragments from DNA both in vitro and in human fibroblasts [21]. However, this could be partly due to the ability of mammalian NER to process undamaged DNA [66]. Regarding their templating properties, clickable pyrimidine nucleotides were investigated as substrates for polymerase chain reaction, which obviously requires good bypass to generate full-length products. C5-modified clickable pyrimidine dNTPs were reported to be rather poor PCR substrates for Taq and Tth polymerases (Family A) but better ones for thermophilic Family B enzymes Pwo, Vent and KOD [67,68].

In the present study, we show that EtU and its analog C8-AlkU with a bulkier C5 substituent are removed in vitro by human DNA glycosylases SMUG1 and MBD4. SMUG1, a bona fide uracil–DNA glycosylase with a preference for U:G mismatches, can also excise pyrimidines with modifications at C5, such as 5-hydroxymethyluracil, 5-formyluracil, 5-carboxyuracil and 5-hydroxyuracil, and even some purines such as xanthine [15,69,70,71,72,73,74]. At the same time, SMUG1 rejects T, its saturated-ring derivatives (dihydrothymine, thymine glycol) and uracil with C5-chloro, -bromo and -iodo substituents [15,71], so it has been proposed that SMUG1 substrates should be able to form direct or water-mediated hydrogen bonds between O^4^ and the group at C5 for the efficient excision of nucleobases bulkier than U [75]. However, as neither EtU nor C8-AlkU are capable of forming such bonds but are excised quite well, the mechanism of base recognition by SMUG1 may need reconsideration. An even wider substrate specificity was reported for MBD4: in addition to being active on T:G and U:G mispairs, it also excises C5-oxidized thymine derivatives and C5-halogenated uracil derivatives [14,16,18,45,76,77]. In the structure of MBD4, the damaged base is everted from DNA into the active site pocket, with the C5 position pointing to a wide cleft, thus allowing for the accommodation of a wide variety of substituents [16,18]. A point to consider about MBD4 is that the enzyme is presumably specific for damaged CpG sites. Although this specificity is thought to be imparted by a separate methyl-CpG-binding domain [18,78], our results show that T and U were rather poorly excised from a non-CpG context (namely, XpC, where X is the modification site). On the other hand, both EtU and C8-AlkU were robustly excised, raising the possibility of BER involvement in their removal. Additionally, some other human DNA glycosylases recognizing pyrimidine lesions, such as TDG, NEIL3 or NTHL1 [41,79,80], could be active against EtU, C8-AlkU or C8-AlkC, but they were not tested here.

Despite the ability of SMUG1 and MBD4 to initiate the BER of EtU and C8-AlkU, the results of our reporter plasmid experiments strongly suggest that they persist in the cells for much longer times than structurally related DNA lesions, U and sUs. The latter is poorly repaired by BER since the phosphorothioate groups inhibit glycosylase, AP endonuclease and AP lyase activities [62,63,64], but it is still removed with an appreciable efficiency by some alternative pathway [34]. However, EtU- and C8-AlkU-modified plasmids produced much higher fluorescence levels 24 h post-transfection. Since in the reporter system that we used, successful repair eliminates the fluorescent signal, we conclude that these modifications are removed inefficiently. One caveat is that, by the reporter system design, the modification is placed opposite to T (the first position of the stop codon), which could prevent its excision by SMUG1 and MBD4. However, other possible repair pathways such as transcription-coupled NER [21] or replication-independent non-canonical mismatch repair [81] should not be affected by this structure. The reasons under the inefficient integral removal of EtU- and C8-AlkU merit further investigation.

DNA polymerases mostly recognized EtU, C8-AlkU and C8-AlkC in the usual Watson–Crick complementarity mode, although misincorporation, especially of dAMP and dGMP opposite to their non-complementary pyrimidines, was evident. While this may seem not too important for short-term metabolic labeling, uncorrected polymerase errors might trigger DNA damage response, which in turn affects replication. Our results also emphasize a need for mutation control if DNA containing clickable nucleotides is used in PCR and/or sequencing techniques such as MINCE-Seq, Hammer-Seq or Double-Click-Seq [82,83,84]. The results with POLβ are especially interesting, since, to our knowledge, this is the first case when this enzyme shows lower insertion fidelity on its preferred substrate, a single-nucleotide gap, compared with the primer–template system. A well-recognized advantage of click DNA labeling is the possibility to achieve high label density [6]; however, in this case, the repair synthesis after the excision of a modification in one strand might pass through a modified nucleotide in the opposite strand and induce misincorporation.

Overall, the main conclusion of our study is rather reassuring: clickable pyrimidine nucleotides, in principle, can undergo repair and be a source of mutations, but the frequency of such events in the cell is not likely to be a considerable source of experimental error under most circumstances. However, more studies are certainly warranted to single out situations when the repair or polymerase misreading of clickable nucleotides might be important.

## Figures and Tables

**Table 1:** Rate constants of cleavage of clickable U derivatives by SMUG1 and MBD4.

Enzyme	Substrate			
	U:G	T:G	EtU:G	C8-AlkU:G
	*k*_2 app_, min^−1^			
SMUG1	>12	(2.3 ± 0.5) × 10^−2^	(2.6 ± 0.1) × 10^−1^	(2.3 ± 0.3) × 10^−2^
MBD4	(2.7 ± 0.4) × 10^−1^	(5.0 ± 0.3) × 10^−1^	2.9 ± 0.2	1.6 ± 0.2

**Table 2:** Steady-state kinetic parameters for dNMP incorporation opposite clickable pyrimidine nucleotides by RBpol and POLβ ^a^.

Enzyme	Template	Incoming	*K*_M_, µM	*k*_cat_, s^−1^	*k*_cat_/*K*_M_, µM^−1^s^−1^	*f*_err_ ^b^	gap/PT ^c^
RBpol	EtU	dATP	5.1 ± 1.1	0.027 ± 0.001	(5.2 ± 1.1) × 10^−3^		
	C8-AlkU	dATP	n/s ^d^	n/s	(1.8 ± 0.1) × 10^−5^		
	C8-AlkC	dGTP	1.2 ± 0.3	0.036 ± 0.001	(3.0 ± 0.8) × 10^−2^		
POLβ	EtU PT	dATP	120 ± 30	0.47 ± 0.04	(3.9 ± 1.1) × 10^−3^		
	EtU gap	dATP	5.5 ± 2.5	0.17 ± 0.01	(3.2 ± 1.4) × 10^−2^		8.1
	EtU gap	dGTP	28 ± 6	0.049 ± 0.002	(1.8 ± 0.4) × 10^−3^	0.056	
	EtU gap	dCTP	56 ± 12	0.025 ± 0.001	(4.5 ± 1.0) × 10^−4^	0.014	
	C8-AlkU PT	dATP	95 ± 32	0.51 ± 0.05	(5.4 ± 1.9) × 10^−3^		
	C8-AlkU gap	dATP	9.0 ± 2.7	0.28 ± 0.01	(3.0 ± 0.9) × 10^−2^		5.6
	C8-AlkU gap	dGTP	27 ± 6	0.022 ± 0.001	(8.1 ± 1.7) × 10^−4^	0.026	
	C8-AlkU gap	dCTP	20 ± 6	0.018 ± 0.001	(9.3 ± 2.6) × 10^−4^	0.030	
	C8-AlkC PT	dGTP	66 ± 19	0.38 ± 0.03	(5.8 ± 1.7) × 10^−3^		
	C8-AlkC gap	dGTP	7.6 ± 1.4	0.24 ± 0.01	(3.2 ± 0.6) × 10^−2^		5.5

^a^ Mean ± s.e.m from three to four independent experiments. ^b^ *f*_err_, frequency of errors, the ratio or *k*_cat_/*K*_M_ values for the incorporation of a non-complementary nucleotide to the complementary one, ^c^ gap/PT, gap preference, the ratio or *k*_cat_/*K*_M_ values for the incorporation into a 1 nt gap substrate over the primer–template (PT) substrate, ^d^ n/s, not saturated; the saturation of the enzyme was not achieved in the used dNTP range; *k*_cat_/*K*_M_ was determined from the linear slope of the *v*_0_ vs. [S]_0_ (dNTP) dependence.

## Data Availability

All data are reported in this paper and the Supplementary Materials.

## Supplementary Materials

The following supporting information can be downloaded at https://www.mdpi.com/article/10.3390/biom14060681/s1, Figure S1: Lack of cleavage of DNA substrates containing C7yn paired with G or A or in single-stranded DNA by DNA glycosylases; Table S1: Oligonucleotides used in this work; Table S2: Optimized reaction conditions for steady-state DNA polymerase kinetics. Original images of Figure 2a–c, Figure S1, Figure 4a,b, Figure 5a–c and Figure 6a,b.
