# Supplementary material for: Repair and DNA Polymerase Bypass of Clickable Pyrimidine Nucleotides
Source: Biomolecules. 2024 Jun 12;14(6):681. doi: 10.3390/biom14060681 (PMC11201982; doi:10.3390/biom14060681)
Supplement: Supplementary file 1 [file biomolecules-14-00681-s001.zip › biomolecules-3031593-original-images.pdf]

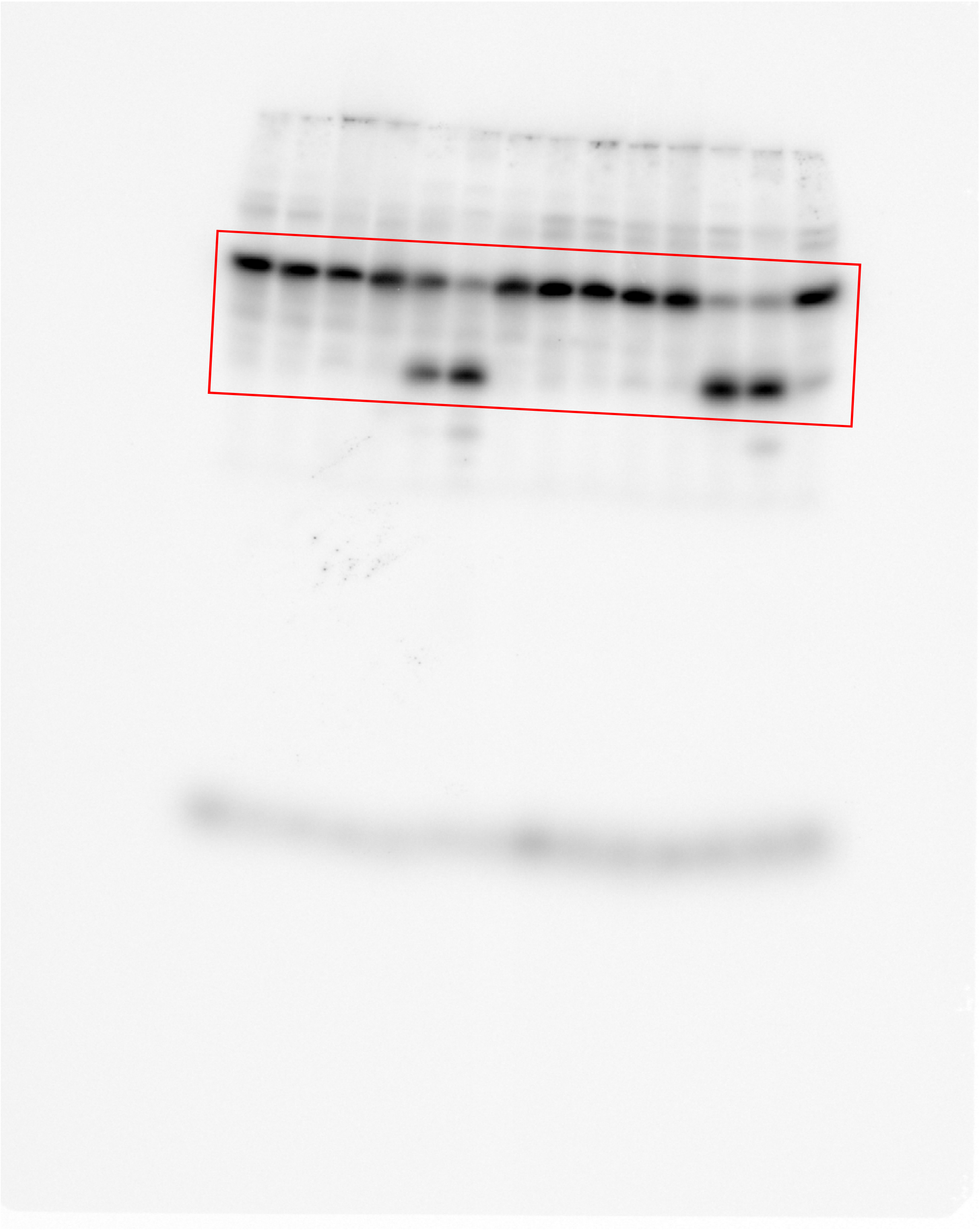

Figure 2a

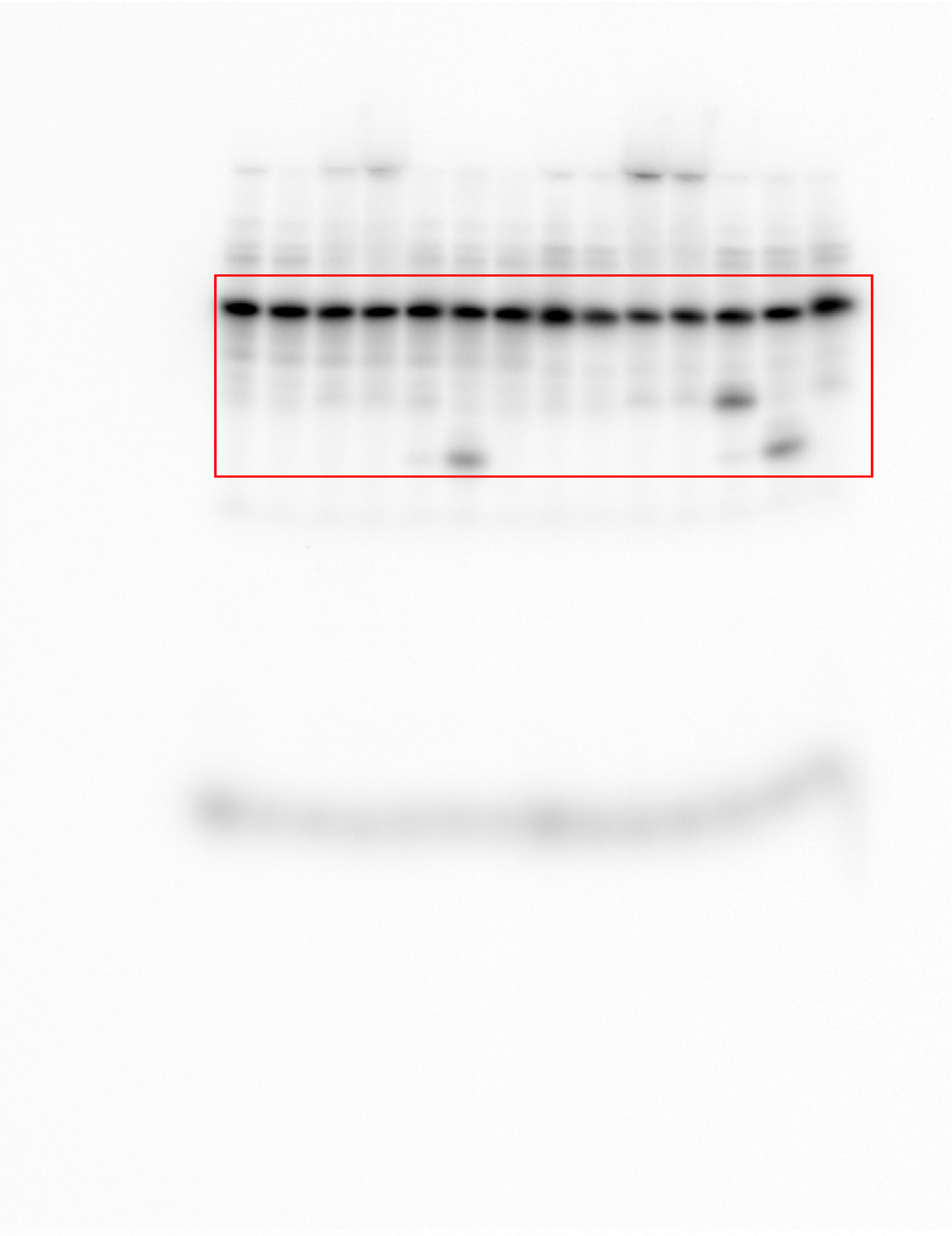

Figure 2b

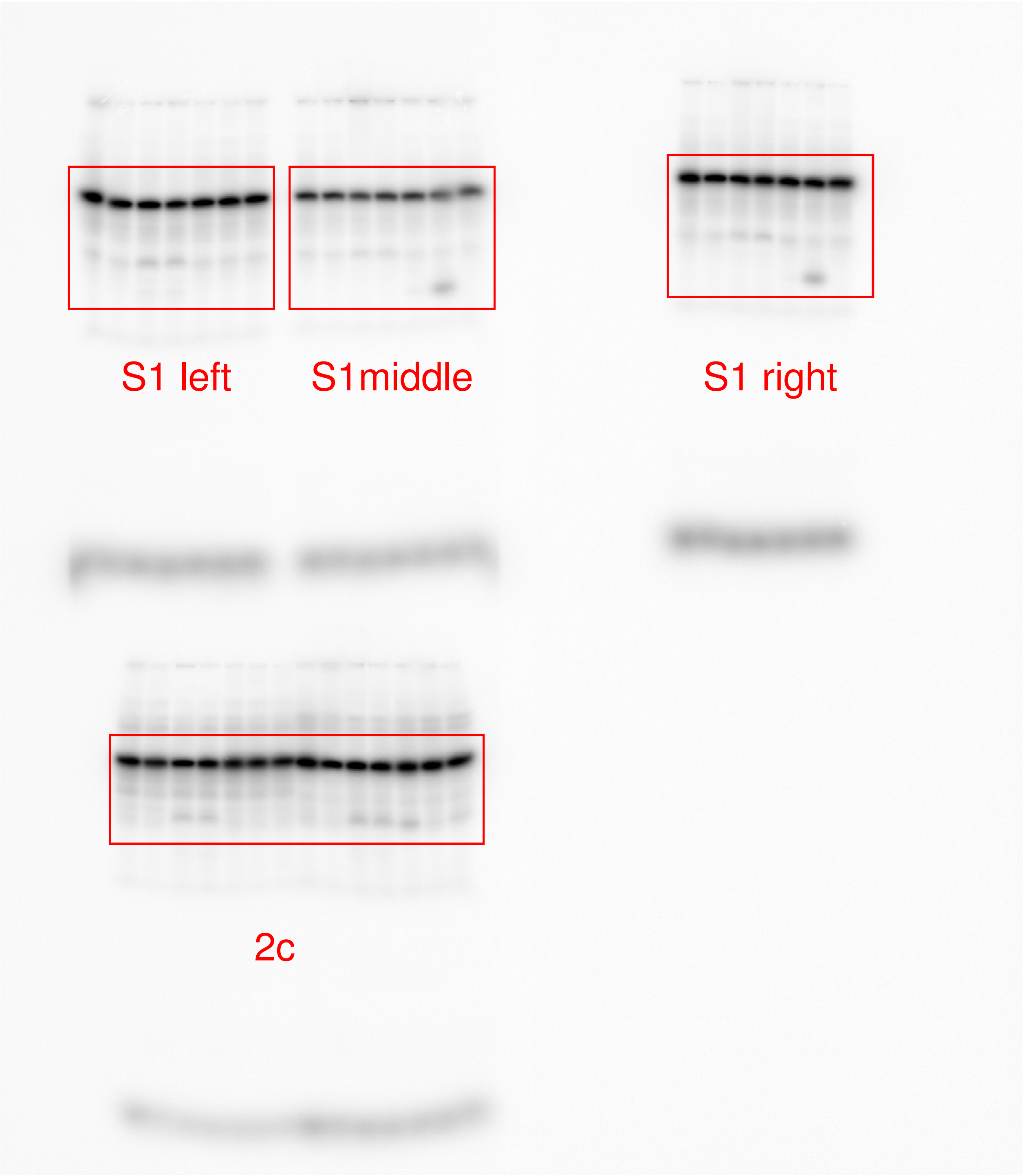

Figure 2c and Supplementary Figure S1

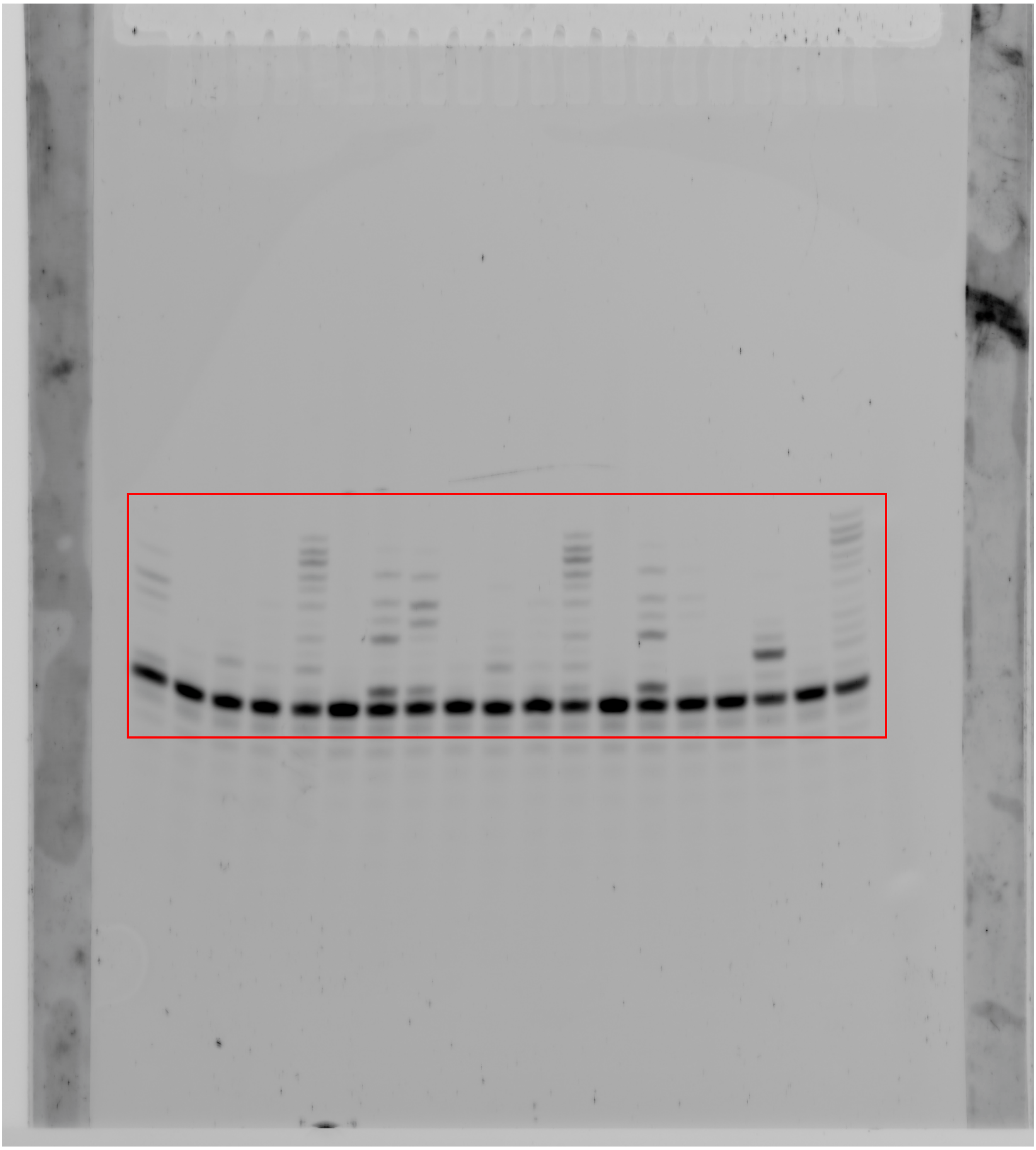

Figure 4a

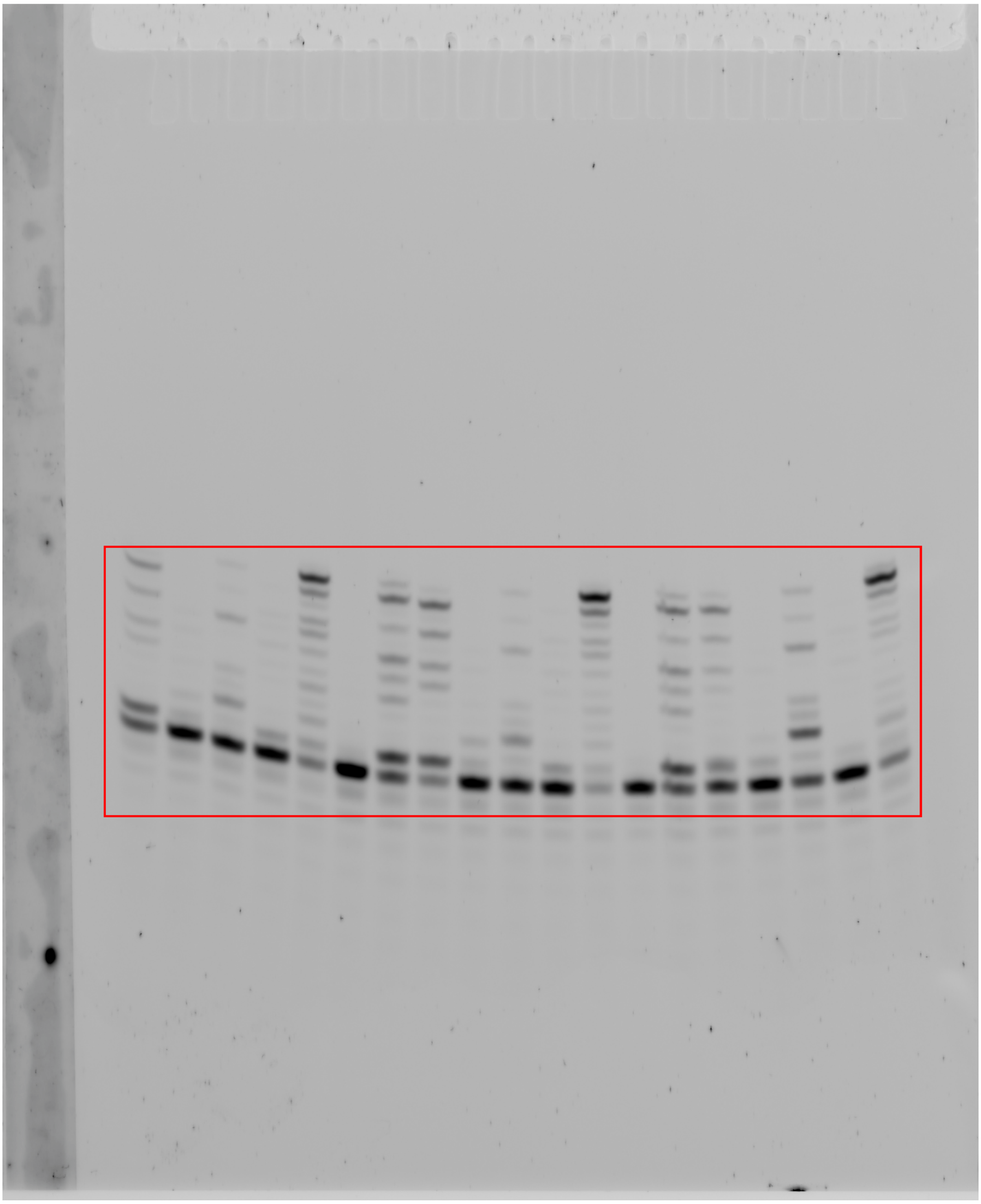

Figure 4b

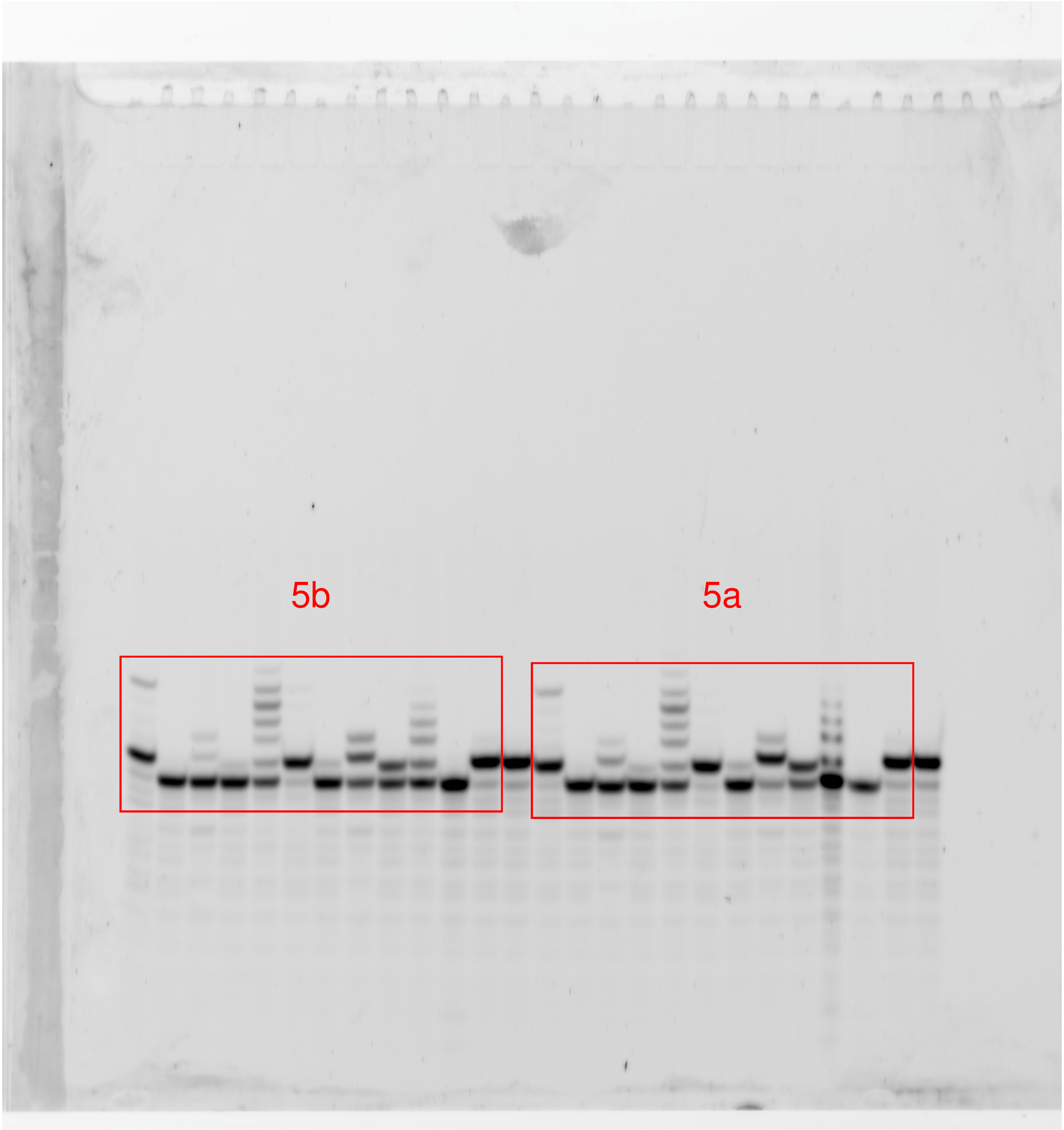

Figure 5a,b

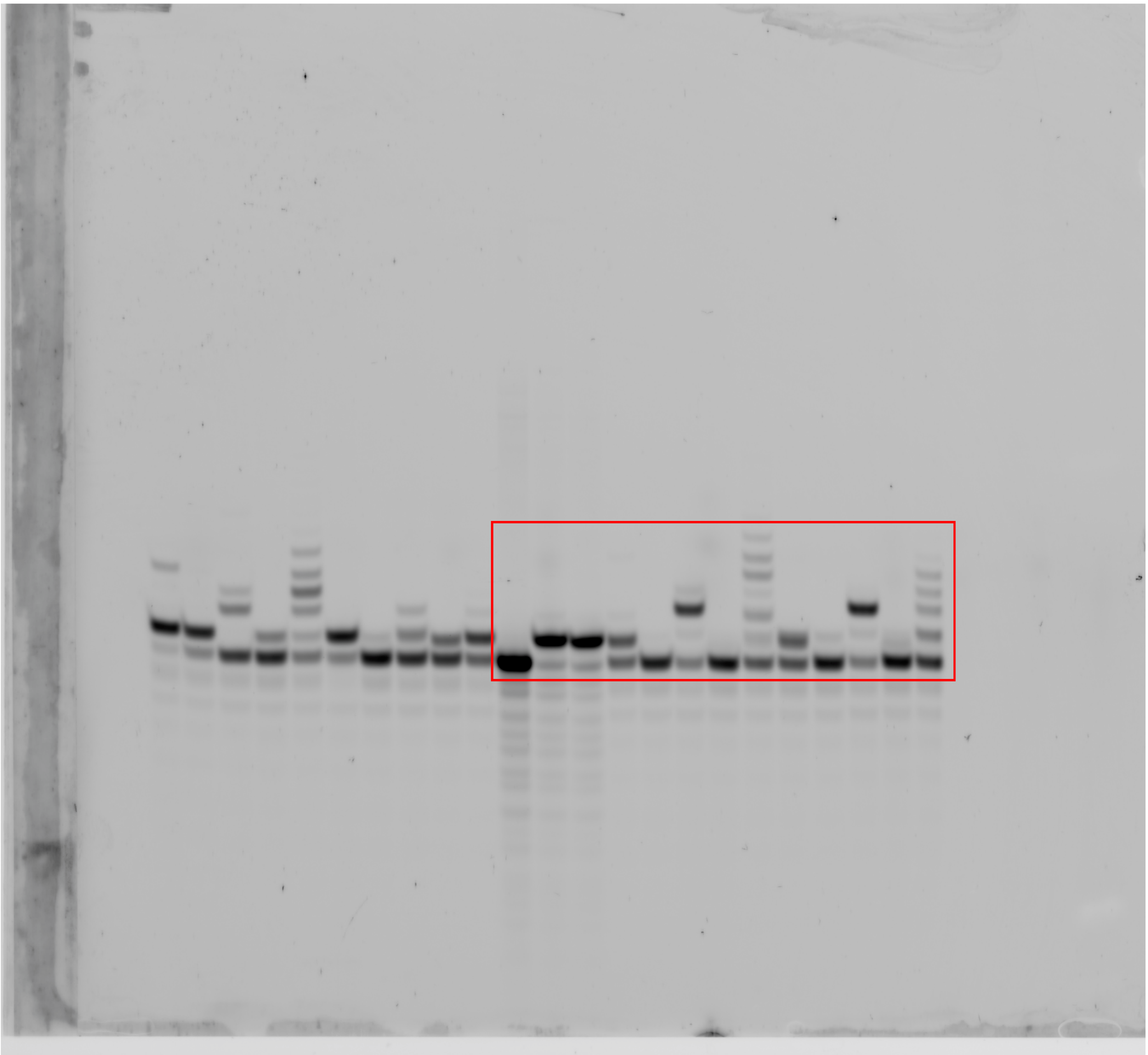

Figure 5c

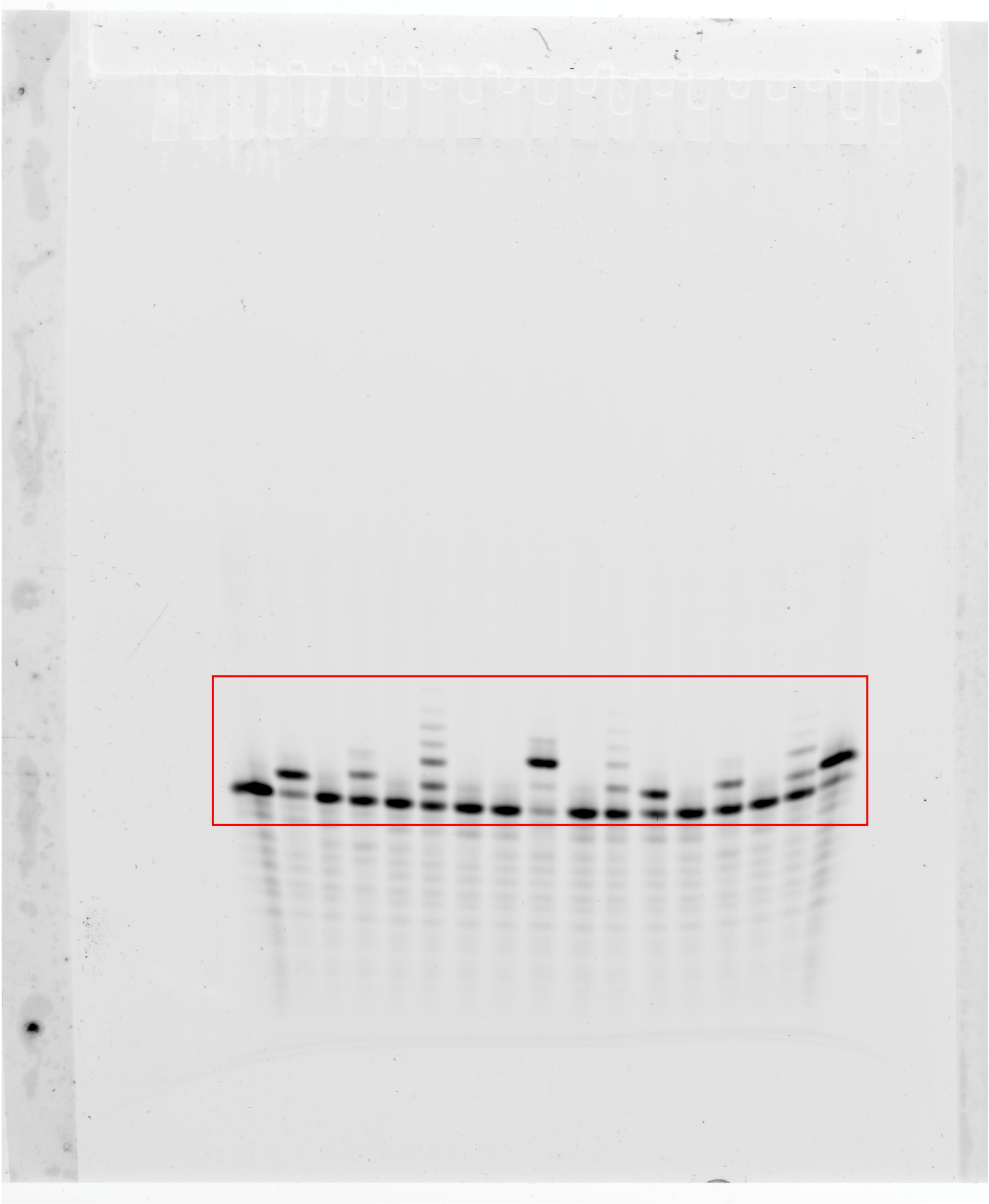

Figure 6a

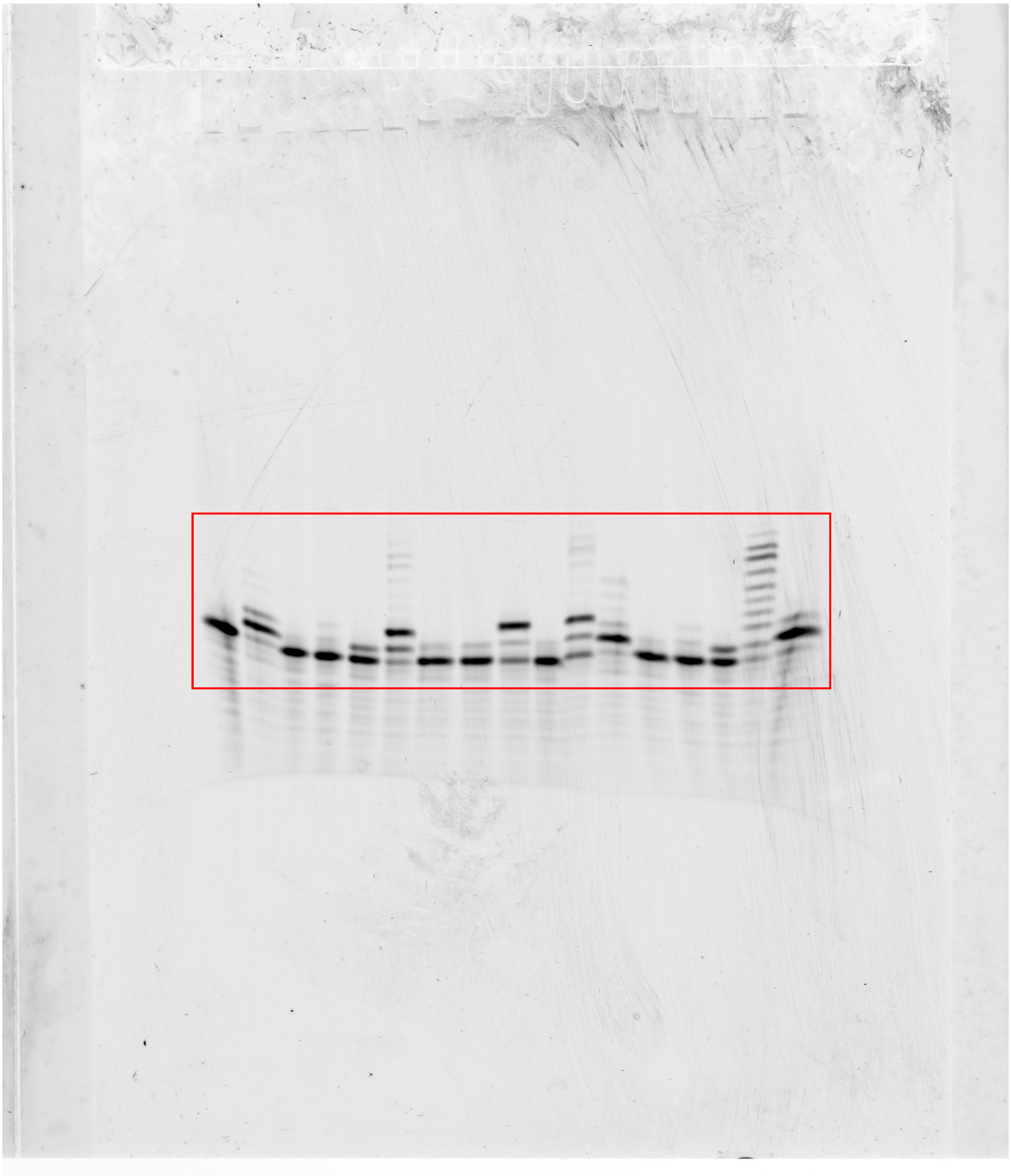

Figure 6b
