# Supplementary material for: Repair and DNA Polymerase Bypass of Clickable Pyrimidine Nucleotides
Source: Biomolecules. 2024 Jun 12;14(6):681. doi: 10.3390/biom14060681 (PMC11201982; doi:10.3390/biom14060681)
Supplement: Supplementary file 1 [file biomolecules-14-00681-s001.zip › biomolecules-3031593-supplementary.pdf]

SUPPLEMENTARY MATERIAL FOR

Repair and DNA polymerase bypass of clickable pyrimidine nucleotides

Anton V. Endutkin [1,*], Anna V. Yudkina [1], Timofey D. Zharkov [1], Alexander E. Barmatov [1], Daria V. Petrova [1], Daria V. Kim [1], and Dmitry O. Zharkov [1,2,*]

[1] Siberian Branch of the Russian Academy of Sciences Institute of Chemical Biology and Fundamental Medicine, 8 Lavrentieva Ave., 630090 Novosibirsk, Russia

[2] Department of Natural Sciences, Novosibirsk State University, 2 Pirogova St., 630090 Novosibirsk, Russia

[*] Correspondence: dzharkov@niboch.nsc.ru (D.O.Z.); aend@niboch.nsc.ru (A.V.E.)

This file contains:

- Supplementary Tables S1–S2
- Supplementary Figure S1
- Supplementary References

Supplementary Table S1. Oligonucleotides used in this work

ID	Sequence (5′→3′)	Modification
DNA glycosylase experiments and DNA polymerase templates		
23EtU	CTCTCCCTTCXCTCCTTTCCTCT	X = 5-ethynyl-U
23C8-AlkU	CTCTCCCTTCXCTCCTTTCCTCT	X = 5-(octa-1,7-diyn-1-yl)-U
23C8-AlkC	CTCTCCCTTCXCTCCTTTCCTCT	X = 5-(octa-1,7-diyn-1-yl)-C
23U	CTCTCCCTTCXCTCCTTTCCTCT	X = U
23T	CTCTCCCTTCTCTCCTTTCCTCT	
c23A	AGAGGAAAGGAGAGAAGGGAGAG	
c23C	AGAGGAAAGGAGCGAAGGGAGAG	
c23G	AGAGGAAAGGAGGGAAGGGAGAG	
c23T	AGAGGAAAGGAGTGAAGGGAGAG	
Non-template oligonucleotides for DNA polymerase experiments		
top12	[Fluo]AGAGGAAAGGAG	[Fluo] = 5(6)-carboxyfluorescein
top10	(p)GAAGGGAGAG	(p) = phosphate [a]
Transcription mutagenesis experiments [b]		
cod18	GCACCTAGTCCGCCCTGA	
temp18A	TCAGGGCGGACTAGGTGC	
temp18U	TCAGGGCGGACTXGGTGC	X = U
temp18sUs	TCAGGGCGGACTXGGTGC	X = 5′,3′-diphosphothio-dU
temp18EtU	TCAGGGCGGACTXGGTGC	X = 5-ethynyl-U
temp18C8-AlkU	TCAGGGCGGACTXGGTGC	X = 5-(octa-1,7-diyn-1-yl)-U

[a] The 5′-terminus was phosphorylated during the synthesis.
[b] The detailed workflow of the reporter plasmid constriction can be found in [1, 2].

Supplementary Table S2. Optimized reaction conditions for steady-state DNA polymerase kinetics

Enzyme	DNA	dNTP	System	Enzyme, nM	dNTP range, µM
RBpol	EtU	dATP	primer–template	1	2–500
RBpol	C8-AlkU	dATP	primer–template	1	10–1000
RBpol	C8-AlkC	dGTP	primer–template	0.5	2–500
POLβ	EtU	dATP	primer–template	0.05	10–750
POLβ	C8-AlkU	dATP	primer–template	0.05	10–750
POLβ	C8-AlkC	dGTP	primer–template	0.1	10–750
POLβ	EtU	dATP	gap	0.1	5–750
POLβ	C8-AlkU	dATP	gap	0.1	10–750
POLβ	C8-AlkC	dGTP	gap	0.1	5–750
POLβ	EtU	dGTP	gap	0.5	10–750
POLβ	EtU	dCTP	gap	1	10–750
POLβ	C8-AlkU	dGTP	gap	1	10–750
POLβ	C8-AlkU	dCTP	gap	1	10–750

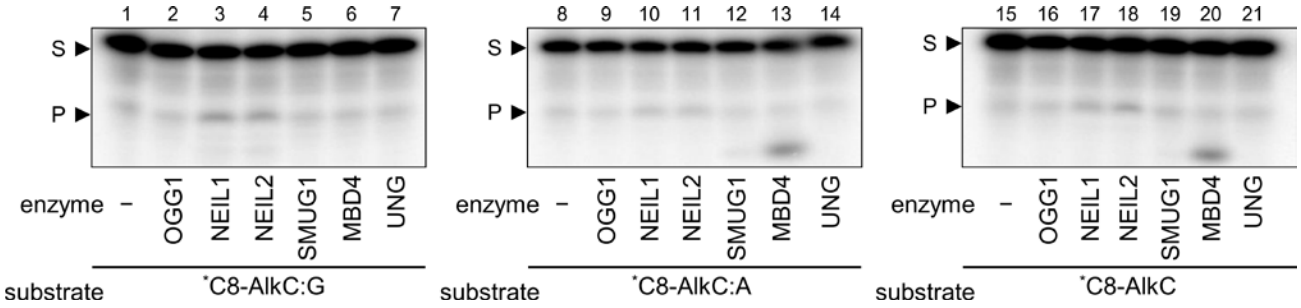

Supplementary Figure S1. Lack of cleavage of DNA substrates containing C7yn paired with G or A or in single-stranded DNA by DNA glycosylases. S, substrate; P, cleavage product. Asterisk indicates the ^{32}P-labeled strand.

Supplementary References

1. Lühnsdorf, B.; Kitsera, N.; Warken, D.; Lingg, T.; Epe, B.; Khobta, A. Generation of reporter plasmids containing defined base modifications in the DNA strand of choice. *Anal. Biochem.* **2012**, *425*, 47–53. 10.1016/j.ab.2012.03.001
2. Kim, D.V.; Diatlova, E.A.; Zharkov, T.D.; Melentyev, V.S.; Yudkina, A.V.; Endutkin, A.V.; Zharkov, D.O. Back-up base excision DNA repair in human cells deficient in the major AP endonuclease, APE1. *Int. J. Mol. Sci.* **2024**, *25*, 64. 10.3390/ijms25010064
